# Safety of Qigong: Protocol for an overview of systematic reviews

**DOI:** 10.1097/MD.0000000000013042

**Published:** 2018-11-02

**Authors:** Yu Guo, Mingmin M. Xu, Yuchang Huang, Meiqi Ji, Zeren Wei, Jialei Zhang, Qingchuan Hu, Jian Yan, Yue Chen, Jiaxuan Lyu, Xiaoqian Shao, Ying Wang, Jiamei Guo, Yulong Wei

**Affiliations:** a School of Acupuncture-Moxibustion and Tuina, Beijing University of Chinese Medicine, Beijing; b Ovation Health Science and Technology Co. Ltd, ENN Group, Langfang; c School of Acupuncture-Moxibustion and Tuina, Chengdu University of Traditional Chinese Medicine, Chengdu; d Department of Ophthalmology, China-Japan Friendship Hospital, Beijing, China.

**Keywords:** adverse event, overview of systematic review, protocol, *Qigong*, safety

## Abstract

**Background:**

Qigong, as one of the essential elements of Traditional Chinese exercises, has been used to improve physical and psychological health and combat diseases in China for thousands of years. In recent years, the beneficial effects of Qigong on different medical conditions are becoming more accepted by both patients and health care providers. Although it is a common impression that Qigong and related therapies are generally safe procedures, but the current understanding of its adverse events is fragmented. Thus, we conducted this overview to synthesize comprehensively existing systematic reviews on adverse events associated with Qigong and related therapies, and our findings can be used to informing clinicians, Qigong practitioner, and patients alike on applying such treatments or interventions in clinical treatment and daily life training mindful manner, and provide a guideline for researchers in future.

**Methods:**

A systematic review of reviews will be performed. A literature search strategy designed by a number of specialists in the fields of Traditional Chinese Medicine (TCM), sports medicine, health information, and Qigong training will be carried out in relevant English and Chinese electronic database. The date range of search will start from inception to the search date. Two reviewers will identify relevant studies, extract data information, and then assess the methodical quality by Assessment of Multiple Systematic Reviews (AMSTAR) tool. Any types of systematic review that summarized adverse effects related to Qigong and related therapies in human will be included. Any safety-related outcomes will be considered as the primary outcomes of this overview. Where objectives from 2 or more reviews overlap, we will assess the causes of any noted discrepancies between reviews. An overall summary of results will be performed using tabular and graphical approaches and will be supplemented by narrative description.

**Results:**

This overview will identify any adverse events associated with nonstandardized Qigong and related therapies procedures based on current relevant literature evidence of safety for Qigong.

**Conclusion:**

Our overview will provide evidence to help synthesize the broad degree of information available on furthering the knowledge, safety, and application of Qigong.

**Ethics and dissemination:**

Formal ethical approval is not required, as this study is an overview based on the published systematic reviews. The result of this overview of systematic reviews will be published in a peer-reviewed journal or disseminated at national and international conferences.

**PROSPERO registration number:**

PROSPERO CRD42018109409

## 1 Introduction

Qigong, translates from Chinese to mean,^[1]^ Qi means vital life-energy that flows in channels (meridians) in the body and Gong means training or cultivation of the Qi.^[2,3]^ Qigong, as a gentle low-impact mind-body aerobic exercise, has been recognized as a “medical” exercise and used to improve physical and psychological health and combat diseases in China for thousands of years.^[3–6]^ The characteristic of Qigong is self-directed and basic components of that include concentration, relaxation, meditation, rhythmic breathing regulation, body posture, and gentle movement.^[5–8]^ The definition can be understood to practice Qigong is to practice the 3 adjustments, and the aim is to achieve the state of oneness by integrating the adjustments.^[9]^ As we know, as one of the essential elements of Traditional Chinese exercises, earliest forms of Qigong make up one of the historic roots of contemporary Traditional Chinese Medicine (TCM) theory and practice.^[3,4,10,11]^ There are hundreds of forms of Qigong exercises developed in different regions of China that have been created by specific teachers, some designed to benefit certain diseases while most others have general health benefits.^[3,9]^ Such as “The Five-Animal Frolics (*Wuqinxi*),” “The Eight-Section Brocades (*Baduanjin*),” “The Six Syllable Formula (*Liuzijue*),” “Muscle/Tendon Changing Classic (*Yijinjing*),” “Five Elements Plam (*Wuxinzhang)*,” “Health Preserving Qigong(*Baojiangong*),” “Post Standing Qigong(*Zhanzhuanggong*),” “Relaxation Qigong(*Fangsonggong*),” “Internal Nourishing Qigong(*Neiyanggong*),” “meditation,” “mindfulness,” “mind concentration,” and“Guolin New Qigong.” According to the philosophy of TCM, Qigong is based on the theory that the body is a small universe where “Qi” circulates, illness or injury disturbs the harmony of vital energy circulation. Qigong is believed to be a method of achieving a harmonious flow of vital energy and regulate the functional activities of meridians and visceral organs.^[3,12–14]^ With regular practice and rehearsal of the structured postures or movements, as well as concentration on mind and breath, practitioners can achieve an efficiency of “body relaxation and mind calm” and *Tian Ren He Yi* (the theory that mankind is an integral part of nature) so as to experience mood stabilization and improved strength and fitness.^[3,15–18]^ From the perspective of western thought and science, this combination of self-awareness with self-correction of the posture and movement of the body, the flow of breath, and stilling of the mind are thought to comprise a state that activates naturally occurring physiological and psychological mechanisms of self-regulatory (self-healing) capacity, stimulating the balanced release of endogenous neurohormones and a wide array of natural health recovery mechanisms, which are seen as affecting the balance and flow of energy, enhancing functionality in the body and the mind, and intently integration of body and mind.^[4,12,19–21]^

Besides, Qigong is an easily adaptable form of aerobic exercise that can be practiced any place and any time and can be learned by almost anyone of any age or physical condition without any special equipment.^[3–6,14,17,22]^ It is widely practiced by Chinese not only to improve their physical health but also to control their emotions, manage their stress or depressive/anxiety symptoms, and enhance overall well-being.^[1,10,14,18,23,24]^ There are many qigong clinics, and in some hospitals, Qigong is integrated with TCM and with conventional western biomedicine. Several complementary medical therapies with some similarities to Qigong are practiced in hospitals in the west and are paid for by insurance. In recent years, existing systematic reviews have examined the clinical evidence of the beneficial effects of Qigong exercise on different medical conditions, such as tumor and cancer,^[25–27]^ hypertension,^[2,28,29]^ diabetes mellitus,^[30–32]^ obesity,^[33,34]^ chronic heart diseases,^[35,36]^ Parkinson's disease,^[37–39]^ dementia,^[40,41]^ chronic fatigue syndrome,^[42–44]^ menopause syndrome,^[45,46]^ insomnia,^[47–49]^ lower back pain,^[50–52]^ chronic obstructive pulmonary disease,^[53–55]^ fibromyalgia,^[56–58]^ metabolic disease,^[20,59]^ osteoarthritis,^[60–62]^ mental disease,^[14,19,63,64]^ and so on.

The wide use of Qigong in clinical treatment and daily life training require continual safety evaluation. In China, although it is a common impression that Qigong and related therapies are generally safe procedures and the risk of receiving Qigong training may be lower, but the question has not yet been definitively investigated. Thus, we conducted this overview of all identifiable peer-reviewed relevant publications and critically examine the safety of Qigong in patients or practitioners receiving regular training. And then, this overview of systematic reviews will provide a comprehensive picture of both the evidence needed to make decisions regarding this topic and the research gaps in this area.

## 2 Objectives

As the goal of this overview, based on the methods for Cochrane overviews, will be designed to synthesize comprehensively existing systematic reviews and then summarize systematically the best current evidence on adverse events associated with Qigong and related therapies, thus our findings can be used to informing clinicians, Qigong practitioner, and patients alike on applying such treatments or interventions in clinical treatment and daily life training mindful manner, and provide a guideline for researchers in future.

## 3 Study methods and analysis

This protocol of overview describes the methods will be performed according to recommendations of the Cochrane Collaboration and “Preferred Reporting Items for Systematic Review and Meta-Analysis Protocols” (PRISMA-P) statement guidelines.^[65]^ This review has been registered on the International prospective register of systematic reviews (PROSPERO), registration number: CRD42018109409. (https://www.crd.york.ac.uk/prospero/display_record.php?RecordID=109409).

### 3.1 Research questions to be addressed

The main purpose of this overview is to evaluate the frequency and type of adverse event occurrences of Qigong and related therapies for all populations. A secondary aim is to evaluate the consistency and quality of *adverse events* monitoring protocols used in the included trials. This study has been designed to answer the following primary research question: *How many kinds of adverse events and what are the main adverse events in Qigong training, besides, what is the incidence of adverse Qigong events in clinical treatment and daily life training? Then on the base of these, how should we avoid and prevent the occurrence of adverse events regarding Qigong and related therapies?* In the context of the review, a series of secondary objectives will also be addressed. These will include assessment of Qigong adverse events and incidence within different practitioner age groups (e.g., teenager, adult, and elderly practitioners), different practitioner sex groups (e.g., male and female), settings (e.g., community, home, school, professional institutions, and hospitals), durations and frequencies of Qigong training (e.g., acute, continuing, and long-term training, or often, once in a while), types or forms of Qigong training (e.g., dynamic Qigong and static Qigong), assessment of the different cited types and causes of preventable adverse Qigong events (both with their corresponding distribution of frequency), and the severity of practitioner outcomes associated with their occurrence. We conclude with recommendations for improving our understanding of the safety of Qigong and related therapies, including guidelines for reporting adverse events in future training of Qigong and related therapies.

### 3.2 Study eligibility criteria

Eligibility criteria have been prepared in terms of the participants: intervention/comparator-outcomes-study design (PICOS) framework, which is helpful and form the basis to establish eligibility criteria, with this additional component, the types of study design. We will consider systematic reviews and primary studies included in those reviews according to the following criteria defined below.

#### 3.2.1 Type of reviews

The current study is designed to be an overview of existing reviews because systematic reviews rather than original trials utilize the widest range of relevant evidence, and thus, any types of systematic review that summarized adverse effects related to Qigong and related therapies in human patients or volunteers, including *Baduanjin Qigong, Wuqinxi Qigong, Liuzijue Qigong, Tai Chi Qigong, Yijinjing Qigong, Meditation Qigong, Mindfulness Qigong, Post Standing Qigong*, *Guo Lin New Qigong Therapy, Relaxation Qigong, Internal Nourishing Qigong, Health Preserving Qigong, and other forms of Qigong*, were considered eligible for this overview. Those reporting on the occurrence of adverse Qigong events where Qigong were adequately administered will not be eligible.

To be included, the systematic reviews must have a primary objective of identifying adverse events instead of investigating its treatment efficacy or effectiveness. However, systematic reviews on adverse effects specifically caused by self-psychosomatic problems, failure to follow the principles of Qigong training, lack of concentration and attention in Qigong training, and were excluded.

We will consider publications to be peer-reviewed systematic reviews as full text as well as any published as abstract only if they were clearly described in the report as being based on an explicit and systematic search strategy of one or more electronic literature databases, clearly specified the review question and methodology with explicit eligibility criteria, involved study selection and data collection by 2 or more reviewers, performed some form of risk of bias appraisal of included studies, provide a systematic presentation and summaries of the characteristics and findings of the included reviews, and synthesized all information using a quantitative or qualitative approach. Review articles not meeting these criteria or animal research and in vitro studies will be also excluded.

#### 3.2.2 Type of population

We had no restriction for the type of patients or practitioner included, as long as they received Qigong or related therapies for the management of physical and psychological wellbeing, or any diseases and symptoms. Thus, we will include systematic reviews that summarizes studies target patients or practitioner receiving various forms of Qigong training intervention from community, home, school, professional institutions, or hospitals and being treated and prevented with Qigong will be sought.

#### 3.2.3 Types of intervention

No specific Qigong intervention is required for a study to be eligible for this overview. Systematic reviews covering all types of Qigong intervention aimed at the management of physical and psychological wellbeing, or any diseases and symptoms will be identified. No restriction on duration and frequency (if applicable) of Qigong training will be imposed.

#### 3.2.4 Types of comparisons

We did not set any restriction on the control treatment or intervention as long as adverse effects of Qigong or related therapies were reported. These Qigong interventions can either be compared with control interventions (standard or usual treatment/care), no intervention (treatment or exercise), or alternative conventional physical exercise such as jogging or walking, and so on.

#### 3.2.5 Types of outcomes

To be included, the systematic review must have a primary objective of identifying adverse events instead of investigating its treatment or intervention efficacy or effectiveness. Any safety-related outcomes will be considered as the primary outcomes of this overview.

Adverse events were further divided into 2 types, “Serious” and “Other (not including Serious).” In accordance with its definition of an adverse event and the definition of Qigong-related adverse events in the previous literature, the following definition of Qigong-related adverse events will be defined^[9]^: “a variety of undesirable experience or any slightly unfavorable and unintended sign, feeling, symptom, physical and mental changes or disease that participants endure during or after treatment or intervention with Qigong training regardless of causal relationship, but are not serious to the point of affecting normal life and work.” And serious adverse events are defined that the event led to serious outcomes such as being life-threatening, permanent damage, require either in-patient hospitalization or the prolongation of hospitalization, results in persistent or significant disability/incapacity or death.^[9]^ Thus, serious adverse events in the Qigong training refer to Qigong “deviation,” also known as “overrunning” of fire and entrance of demons, or deviation for short, which is the serious negative somatic or mental reactions in the course of practicing Qigong. Deviation is represented by functional, psychological, emotional, or behavioral disorders that affect the practitioner's normal life or work and is unlikely to disappear spontaneously. Qigong deviation differs from adverse event that do not interfere with the activities of daily and will mostly disappear spontaneously or be relieved by proper medical intervention.^[9]^

After pre-retrieval and repeated discussion, the range of Qigong-related adverse events include headache, dizziness or vertigo, distension of head, tinnitus, stuffiness in the chest and worsening shortness of breath, heart-pounding or palpitations, muscular soreness or pain, and so on. At the same time, Qigong deviation occurs with a variety of serious negative physiological or psychological changes and symptoms, which can be divided into 2 categories: somatic symptoms (e.g., compression at the top of the head, difficulty in breathing, emission or spermatorrhea, shaking in the arms or legs, profuse cold perspiration of whole body, or intensified and strange ceaseless body and limb movement due to Qi disorder, etc) and mental and emotional symptoms (e.g., neurasthenia, affective disorder, disorder of self-consciousness, hallucination and paranoia, or psychological stress, etc).

### 3.3 Literature search and search strategy

A purposive literature search strategy has been established with the assistance and reviewed of a number of experts in the fields of TCM and sports medicine. The health information specialist and Qigong specialist will be consulted for the development of the search strategies, and this person will help for performing the searches.

Two reviewers will independently conduct sensitive search for eligible systematic reviews through the following relevant electronic databases, such as PubMed Database, Embase Database, Cochrane Library, Web of Science database, Medline, Chinese BioMedical Literature Database, China National Knowledge Infrastructure (CNKI), China Science and Technology Journal database (VIP), and Wanfang Data Chinese database without study type and publication (publication date or publication status) restrictions of systematic review. The date range of search will start from inception to the search date. We will also search for the following sources, which contain systematic reviews and overviews of publications: Database of Abstracts and Reviews (DARE), Health Technology Assessment (HTA) database, TRIP Database, PDQ-Evidence, Epistemonikos, and Health Systems Evidence. The search will be limited to the English and Chinese language literature. This electronic search will be supplemented by a search for unpublished, ongoing, or recently completed systematic reviews in PROSPERO. In addition, we will also conduct hand searches in the reference lists of all included systematic reviews that might meet inclusion for the current overview.

Structured search strategies will be developed using the thesaurus terms of each database and targeting the “title” and “abstract” fields. We will conduct searches in electronic databases using a combination of free text keywords and Medical Subject Heading (MeSH) terms; as we were concerned that most articles poorly report adverse events and are poorly indexed, we decided not to combine search terms for adverse events at the cost of sensitivity. The following search terms were used: Qigong, Ch’i Kung, Qigong therapeutics, Qigong exercise, traditional Chinese exercise, complementary therapies, mind–body exercise, mind-body therapies, breathing exercises, breathing training, breathing technique, respiratory training, respiratory exercise, physical therapy modalities, exercise movement techniques,

Five-Animal Frolics, *Wuqinxi*, Eight-Section Brocades, *Baduanjin*, Six Syllable Formula, *Liuzijue*, Muscle Changing Classic, Tendon Changing Classic, *Yijinjing*, Five Elements Plam, *Wuxinzhang*, Health Preserving Qigong, *Baojiangong*, Post Standing Qigong, *Zhanzhuanggong*, Relaxation Qigong, *Fangsonggong*, Internal Nourishing Qigong, *Neiyanggong*, meditation, mindfulness, mind concentration, *Guolin* New Qigong, Tai Chi, *Taiji*, *Taijichuan*, adverse event, adverse reaction, adverse effect, adverse experience, Qigong deviation, overrunning of fire and entrance of demons, overrunning of fire, entrance of demons, adverse health care event, incident, accident, complication, side effect, error, safe, safety, risk, and an in-depth list of text words given the nature of varying terminology in this area. The equivalent search terms will be translated into Chinese while searching in the Chinese databases. An example of a search strategy in PubMed is presented in Table 1. This strategy will be adapted and refined according to the specificities of the other databases.

**Table 1:**
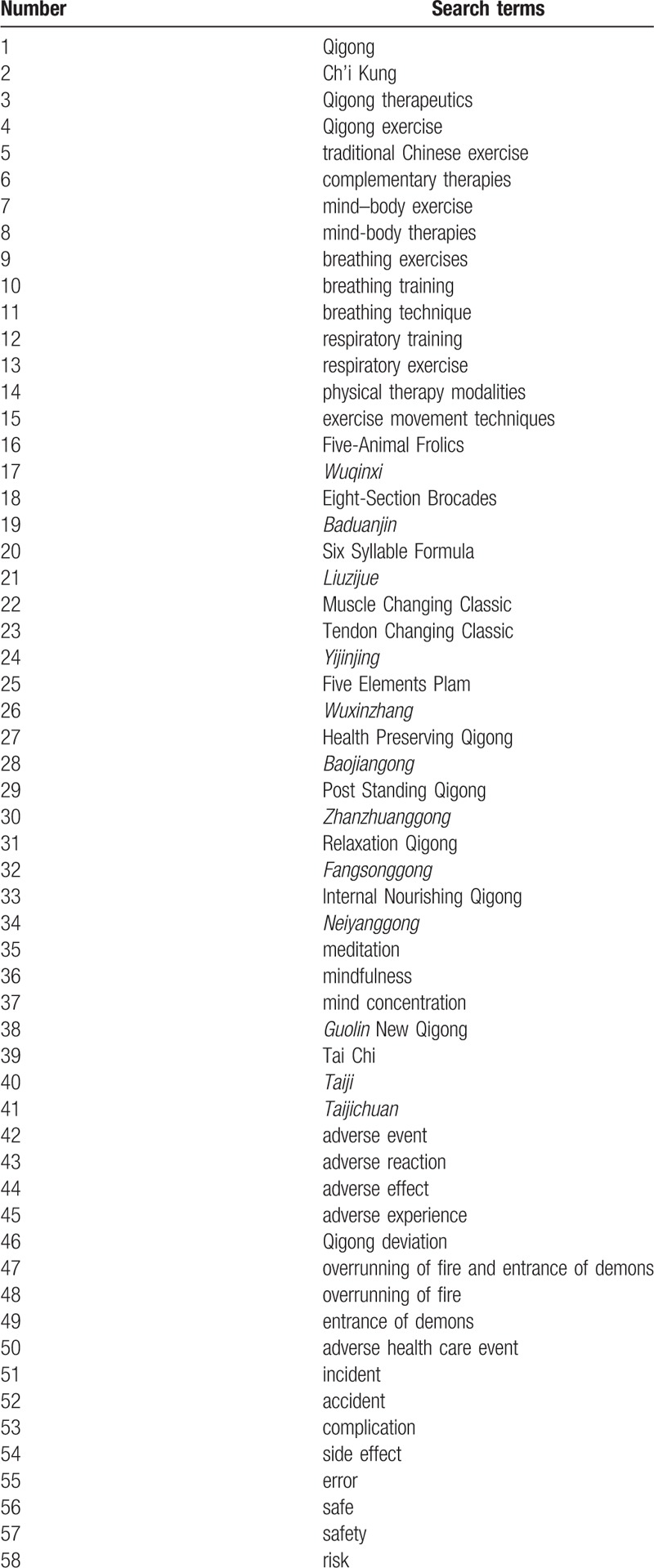
Search strategy for the PubMed database.

### 3.4 Data collection and analysis

The methodology for data extraction and synthesis for this overview will be based on the guidance from PRISMA-P^[65]^ statement and the Cochrane Handbook of Systematic Reviews of Interventions.^[34]^ The chapters give criteria for conducting overviews of systematic reviews.

#### 3.4.1 Process of study selection

**Figure 1:**
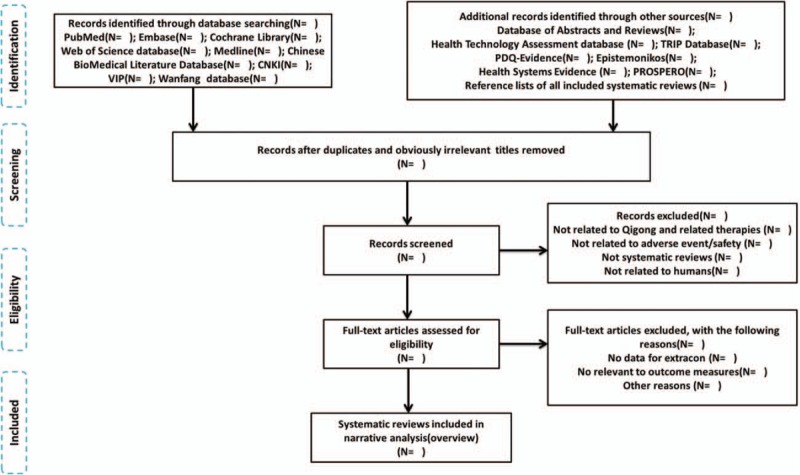
Flowchart of literature selection on systematic reviews of Qigong-related adverse events.

One reviewer will download all of the reviews and will remove any obviously irrelevant titles. Following removal of duplicate material, 2 reviewers will independently screen the search output for results (based on keywords, abstract, and title) of the remaining systematic reviews from the literature search described above in order to assess their eligibility for inclusion in this overview. After initial selection, all citations judged potentially eligible systematic reviews or systematic review protocols will then be further obtained and screened in full-text copies of reports to assess eligibility for final inclusion in the overview. Criteria for inclusion will be based on the type of studies, type of participants, type of interventions, and type of outcome measures. If any ongoing or unpublished study is identified, we will contact the corresponding author for information on the current status of the systematic review (ongoing vs completed) and whether any preliminary data may be included in our overview. Any discrepancies in the inclusion of abstracts or full-text articles will be resolved by discussion and reaching a consensus. If a consensus cannot be reached, consultation of a third member of the review team where necessary. In case of lack of consensus, a third author (TT) will arbitrate. Reviewers will not be blinded to journal titles, study authors, or institutions. Both stages of screening will be preceded by a piloting exercise to ensure that reviewers have a similar understanding of the eligibility criteria. A flow diagram will be presented to describe the process of study selection (Fig. 1).

#### 3.4.2 Data collection and extraction

According to the inclusion, 2 independent reviewers will summarize all included reviews and perform data extraction from the included systematic reviews using an electronic standardized spreadsheet data extraction form to record descriptive characteristics of included reviews. We will extract the following information from the included systematic reviews: features of the review(first author name and institutions, country/countries of origin, journal title, year of publication, review title, date of last search, number of included studies and participants, source of financial support (if any), registration details of the review protocol, if applicable, type of included primary studies, type of adverse event and causality, number of cases, qualification of Qigong trainers); characteristics of population demographics and setting (e.g., healthy people, patient with different diseases, age, gender, total sample size, health condition, primary disease, prognosis, community, home, school, professional institutions, and hospitals), type of interventions (e.g., types or forms, durations and frequencies of training), comparisons(e.g., standard or usual treatment/care, no treatment or exercise, or alternative conventional physical exercise), description of results and conclusions that are relevant to our overview question in adverse event (follow-up period, details about the specific aspects of adverse event, the way outcomes have been measured, different types of outcomes), the key findings, method of assessing quality of studies, reported limitations of review, and the likelihood of causality between the event and acupuncture was assessed in each individual case. We will systematically synthesize the individual studies included within all identified reviews to explore whether any reviews covered the same studies. If an overlap between reviews is identified, 2 overview authors will discuss the overlap with consideration of each review question, comparisons explored, and date of the last search and key aspects of methodological quality (e.g., types of studies included, risk of bias assessment). We will use these details to reach agreement regarding which data from which review comparisons should be included within the overview. Any overlaps between included reviews or comparisons will be transparently reported. Any disagreements arising during the data extraction process will be resolved by discussion and consensus involving the 2 reviewers or will involve a third review author as needed to establish consensus in the presence of disagreements. In case of lack of consensus, a third author (MQJ) will arbitrate, if needed. If any information is missing or incomplete, we will try to contact the review authors. If any data cannot be obtained, then the review will be recorded as an “Included review” without data. To ensure consistency, we will conduct calibration exercises before the review. A data collection form will be drafted and piloted on a small number of studies and discussed among the team to incorporate any necessary refinements before completion of data collection from all relevant studies.

#### 3.4.3 Certainty of evidence in included reviews

We will report the certainty of evidence as assessed by the systematic review authors. If the systematic review authors did not assess the certainty of the evidence, we will assess the certainty of the evidence reported by the review authors using the Grading of Recommendation Assessment, Development, and Evaluation (GRADE) approach as outlined in the GRADE handbook.^[66]^

#### 3.4.4 Quality assessment/methodological quality of included reviews

Two reviewers will independently evaluate the methodological quality/risk of bias for each included systematic review that meets the eligibility criteria, using validated Assessment of Multiple Systematic Reviews (AMSTAR) measurement tool.^[67,68]^ This is most commonly used to assess the quality of systematic reviews included in overviews. AMSTAR includes 11 items, with each of the 11 criteria given a rating of “yes” (definitely done), “no” (definitely not done), “can’t answer” (unclear if completed), or “not applicable” based on information provided by the systematic reviews on which reviewers put a score of 1 point when the criterion is met. Each systematic review will be assigned to 1 of 3 quality levels (0–3 points—low quality, 4–7 points —medium quality, and 8–11 points—high quality). Disagreements between assessors were discussed to reach consensus, and where this is not achieved, arbitration by a third review author (MQJ) will be sought.

#### 3.4.5 Dealing with missing data

Reasons for missing data will be recorded by the original reviews. If the original reviews included this detail, we will try our best to obtain requisite information by contacting the corresponding author of the referenced articles for the missing data whenever possible. If the missing data cannot be obtained, we will report the number of studies that performed the analysis based on the available data to decrease the potential influence of the missing data. The potential impact of the effect of missing data on the final findings of the overview will be addressed in the discussion.

#### 3.4.6 Data synthesis

Adverse effects of Qigong and related therapies were narratively reported of the relevant results for the individual systematic reviews. To summarize findings, a descriptive approach will be performed that will provide a series of summary tables to characterize key features, quality assessment, and major conclusions, and variations of the research, supplemented with graphics to highlight diversity and enhance the clarity in study results and other aspects. This will also include a focused effort to map gaps between reviews in relation to many aspects of adverse Qigong events described. For reviews addressing the same objectives and endpoints, their findings will be compared. In exploring the rationale for variations in findings between the reviews, several strategies will be employed. First, a comparison of review methods will be performed in relation to eligibility criteria (i.e., assessment of variations in criteria used to identify eligible patients or practitioners, study designs, and endpoints of interest), literature search details (dates, databases, and key differences in strategies employed, language restrictions employed), endpoint definitions used, statistical approaches to meta-analysis (if performed), and rigor of review methods (as reflected by variations in AMSTAR assessments and other aspects of study methodology). Second, the evidence base included in different reviews will be evaluated in terms of their degree of overlap; this will involve comparison of date ranges of studies covered by the review, the numbers of studies and volunteers across reviews, and development of a citation matrix to establish the similarity of included study lists. Lastly, comparison of review findings (e.g., meta-analytic findings regarding pooled incidence rates or other related measures) and conclusions drawn by review teams will also be performed. For studies examining the same Qigong interventions, we will state whether the reported conclusions are concordant.

### 3.5 Sensitivity analysis

If applicable, we will conduct sensitivity analysis and summarize the quality of the evidence in relation to the most important outcomes by using the GRADE approach.^[67]^

### 3.6 Subgroup analysis

Depending on sufficiency of reviews, we will explore subgroup analyses according to types or forms of Qigong training, durations and frequencies of Qigong training, settings, different practitioner age groups, different practitioner sex groups, assessment of the different cited types, causes of preventable adverse Qigong events, and so on.

## 4 Ethics and dissemination

The result of this overview of systematic reviews will be published in a peer-reviewed journal or disseminated at relevant conferences presentations. Formal ethical approval is not required because we will search and evaluate only existing sources of literature. Due to the paucity of related publications in the field, this review article will, by adding more recent studies into the analysis, provide more robust evidence of safety of Qigong to clinicians, Qigong practitioner, and patients who apply for such treatments or interventions in clinical treatment and daily life training for the management of physical and psychological wellbeing, or any diseases and symptoms.

## 5 Discussion

In China, Qigong and related therapies have been as modality of treatment for various ailments and medical diseases.^[1,3,4,6]^ It is said that the popularity of Qigong and related therapies is partially attributed to its convenience and safety, and in some oriental countries, Qigong and related therapies are usually conducted by Qigong professionals. However, this kind of intervention is not entirely risk-free, where adverse events, such as distension of the head, palpitation, shortness of breath, hypochondriac distension, muscular soreness or pain, self-feeling of Qi leakage through perineum or anus, intensified and strange ceaseless body and limb movement due to Qi disorder, neurasthenia, affective disorder, and hallucination and paranoia are also reported in the literature^[3,9,69–73]^; meanwhile, when applying Qigong and related therapies to special populations with psychiatric disorder, particularly patients suffering from severe schizophrenia, mania, obsessive-compulsive disorder, unwanted adverse reactions, or Qigong “deviation” could have occurred.^[9,70–73]^ In addition, people who have a family history or personal complications when Qigong and related therapies are implemented, as improper or overexuberant practice may induce further occurrence of psychotic episodes. On the contrary, people who do not suffer psychosis but present with personality disturbance, eccentric conduct, and irrational thinking are not suitable for Qigong and related therapies, because they are at a high risk of trigger-undesired Qi deviation (physical or more often emotional disorientation) during Qigong practice.^[9,70–73]^ Both minor and serious adverse events refer to physical and mental changes or special sensations can occur during Qigong training and they could last some while after practicing.^[3,9,69–73]^ Many publications have reported these adverse events, but they are hard for clinicians or practitioners to digest, as they were written in inconsistent formats. To minimize potential adverse effects and harms caused by Qigong and related therapies, practitioners need to strictly follow standardized procedures of Qigong and related therapies administration as well as fully understand the potential adverse events associated with it. It is important to assess the safety of Qigong and related therapies in clinical practice and daily life training. Different from other traditional therapies such as acupuncture,^[74,75]^ moxibustion,^[76]^ massage,^[77]^ and cupping,^[78,79]^ whose safety is well analyzed in surveys and/or overview of systematic reviews. Currently, despite preliminary scoping of the literature in this area suggests that there exist a number of review articles that have sought to characterize adverse events associated with Qigong and related therapies in different populations and settings,^[69–73]^ there is no overview in existing literature synthesizing the information provided by systematic reviews and meta-analyses on safety or adverse events associated with Qigong and related therapies, and evidence on the safety of Qigong and related therapies have not been clearly established to date. In such situations, this overview serves as an important step toward furthering the knowledge, safety, and application of Qigong. The overview may inform practitioners around the world about and modify the way they practice Qigong, given that many Qigong practitioners and masters may not be fully aware of the full breadth and depth of risk their training can pose. The aim of this study was to evaluate the type and frequency of Qigong and therapies related to adverse events, to identify any avoidable adverse events associated with nonstandardized Qigong and related therapies procedures, and to provide recommendations for improving our understanding of the safety of Qigong and related therapies, including guidelines for reporting adverse events in potential future research.

Some limitations of this overview should be noted. Due to language barriers, our study only included systematic reviews published in the English language, so a language bias may exist. Future studies might include other languages for a better global estimate of adverse event of Qigong training reporting. Second, because of the small number of studies as well as the heterogeneity of both interventions and controls, our study only employed descriptive statistics and narrative summaries of adverse event reports. As the literature evolves, future studies with more formal meta-analyses for assessing relative harms of Qigong when compared with other control interventions may be helpful. Finally, most of systematic reviews only included adverse events reported in randomized trials. And data from audits and cross-sectional studies, especially of longer-term practitioners, as well as uncontrolled longitudinal studies may better inform long-term safety.

## Acknowledgments

The authors would like to deeply acknowledge Professor Tianjin Liu and Haibo Zhang from BUCM, Professor Qing Tang and Weibo Zhang from Ovation Health Science and Technology Co. Ltd, ENN Group for providing valuable suggestions to conduct this overview.

## Author contributions

YG and MMX, YCH and YLW contributed to conceived the idea of research, developed the search strategy, and drafted the manuscript. JLZ and QCH critically revised the manuscript and provided valuable advice on the protocol. YG is in charge of coordination and direct implementation. YLW is responsible for monitored the process of overview. YG and MMX will screen the titles, abstracts, keywords of all retrieved records, and extract data independently. ZRW and JY will assess the risk of bias independently. YC and JXL will deal with the missing data. YW and JMG will conduct statistical analysis, XQS and MQJ will arbitrate any disagreements in the review. All review authors approved the publication of the protocol. All authors participated in the protocol design, commented on drafts of this paper, and read and approved the publication of the final manuscript.

**Conceptualization:** Yu Guo, Mingmin Xu, Yuchang Huang, Yulong Wei.

**Data curation:** Jialei Zhang, Yue Chen, Xiaoqian Shao.

**Formal analysis:** Jian Yan, Ying Wang, Jiamei Guo.

**Investigation:** Yu Guo, Meiqi Ji, Zeren Wei.

**Methodology:** Yu Guo, Mingmin Xu, Meiqi Ji.

**Project administration:** Yu Guo, Mingmin Xu, Meiqi Ji, Yulong Wei.

**Supervision:** Yu Guo, Mingmin Xu, Yulong Wei.

**Validation:** Mingmin Xu, Qingchuan Hu, Jiaxuan Lyu.

**Visualization:** Yu Guo, Mingmin Xu, Yuchang Huang, Meiqi Ji, Zeren Wei, Jialei Zhang, Qingchuan Hu, Jian Yan, Yue Chen, Jiaxuan Lyu, Xiaoqian Shao, Ying Wang, Jiamei Guo, Yulong Wei.

**Writing – original draft:** Yu Guo, Mingmin Xu.

**Writing – review & editing:** Yuchang Huang, Meiqi Ji, Yulong Wei.

Yu Guo: orcid 0000-0002-1752-1254.

## References

[1] Jahnke R Larkey L Rogers C A comprehensive review of health benefits of qigong and tai chi. Am J Health Promot 2010;24:e1–25. 10.4278/ajhp.081013-LIT-248 PMC3085832 20594090

[2] Xiong X Wang P Li X Qigong for hypertension: a systematic review. Medicine 2015;94:e352. 25569652 10.1097/MD.0000000000000352 PMC4602820

[3] Mccaffrey R Fowler NL Qigong practice: a pathway to health and healing. Holist Nurs Pract 2003;17:110–16. 12701998 10.1097/00004650-200303000-00006

[4] Matos LC Sousa CM Gonçalves M Qigong as a traditional vegetative biofeedback therapy: long-term conditioning of physiological mind-body effects. Biomed Res Int 2015;2015:531789. 26137485 10.1155/2015/531789 PMC4475564

[5] Tsang HWH Cheung L Lak DCC Qigong as a psychosocial intervention for depressed elderly with chronic physical illnesses. Int J Geriatr Psychiatry 2002;17:1146–54. 12461764 10.1002/gps.739

[6] Horowitz S Evidence-based health benefits of qigong. Altern Complement Ther 2009;15:178–83.

[7] Xin L Miller YD Brown WJ A qualitative review of the role of Qigong in the management of diabetes. J Altern Complement Med 2007;13:427–33. 17532735 10.1089/acm.2006.6052

[8] Chang PS Knobf MT Funk M Feasibility and acceptability of qigong exercise in community-dwelling older adults in the United States. J Altern Complement Med 2018;24:48–54. 28708414 10.1089/acm.2017.0096

[9] Liu TJ Zhang WC Traditional Chinese Medicine Qigong. Beijng: China Press of Traditional Chinese Medicine; 2016.

[10] Zou L Sasaki JE Wang H A systematic review and meta-analysis Baduanjin Qigong for health benefits: randomized controlled trials. Evid Based Complement Alternat Med 2017;2017:4548706. 28367223 10.1155/2017/4548706 PMC5359459

[11] Fong SS Ng SS Luk WS Effects of qigong exercise on upper limb lymphedema and blood flow in survivors of breast cancer: a pilot study. Integr Cancer Ther 2014;13:54–61. 23749481 10.1177/1534735413490797

[12] Tsang HW Fung KM A review on neurobiological and psychological mechanisms underlying the anti-depressive effect of qigong exercise. J Health Psychol 2008;13:857–63. 18809635 10.1177/1359105308095057

[13] Chang PS Knobf MT Funk M Feasibility and acceptability of Qigong exercise in community-dwelling older adults in the United States. J Altern Complement Med 2017;24:48–54. 28708414 10.1089/acm.2017.0096

[14] Wang CW Chan CH Ho RT Managing stress and anxiety through qigong exercise in healthy adults: a systematic review and meta-analysis of randomized controlled trials. BMC Complement Altern Med 2014;14:8. 24400778 10.1186/1472-6882-14-8 PMC3893407

[15] Tsang HW Tsang WW Jones AY Psycho-physical and neurophysiological effects of qigong on depressed elders with chronic illness. Aging Mental Health 2013;17:336–48. 23072658 10.1080/13607863.2012.732035

[16] Chan AW Lee A Lee DT Evaluation of the sustaining effects of Tai Chi Qigong in the sixth month in promoting psychosocial health in COPD patients: a single-blind, randomized controlled trial. Sci World J 2013;2013:425082. 10.1155/2013/425082 PMC3824309 24282383

[17] Vergeer I Bennie JA Charity MJ Participation trends in holistic movement practices: a 10-year comparison of yoga/Pilates and t’ai chi/qigong use among a national sample of 195,926 Australians. BMC Complement Altern Med 2017;17:296. 28587599 10.1186/s12906-017-1800-6 PMC5461749

[18] Ng BH Tsang HW Psychophysiological outcomes of health qigong for chronic conditions: a systematic review. Psychophysiology 2010;46:257–69. 10.1111/j.1469-8986.2008.00763.x 19170945

[19] Wang F Man JK Lee EK The effects of qigong on anxiety, depression, and psychological well-being: a systematic review and meta-analysis. Evid Based Complement Alternat Med 2013;2013:152738. 23401706 10.1155/2013/152738 PMC3557628

[20] Anderson JG Taylor AG The metabolic syndrome and mind-body therapies: a systematic review. J Nutr Metab 2011;2011:276419. 21773016 10.1155/2011/276419 PMC3136147

[21] Lee MS Lee MS Kim MK Qi-training (qigong) enhanced immune functions: what is the underlying mechanism? Int J Neurosci 2005;115:1099–104. 16040353 10.1080/00207450590914347

[22] Kemp CA Qigong as a therapeutic intervention with older adults. J Holist Nurs 2004;22:351–73. 15486154 10.1177/0898010104269313

[23] Oh B Butow P Mullan B A critical review of the effects of medical Qigong on quality of life, immune function, and survival in cancer patients. Integr Cancer Ther 2012;11:101–10. 21715370 10.1177/1534735411413268

[24] Chow YW Tsang HW Biopsychosocial effects of qigong as a mindful exercise for people with anxiety disorders: a speculative review. J Altern Complement Med 2007;13:831–9. 17983339 10.1089/acm.2007.7166

[25] Husebø A Husebø T Quality of life and breast cancer: how can mind–body exercise therapies help? An overview study. Sports 2017;5:79. 10.3390/sports5040079 PMC5969039 29910438

[26] Wayne PM Lee MS Novakowski J Tai Chi and Qigong for cancer-related symptoms and quality of life: a systematic review and meta-analysis. J Cancer Surviv 2018;12:256–67. 29222705 10.1007/s11764-017-0665-5 PMC5958892

[27] Carlson LE Zelinski E Toivonen K Mind-body therapies in cancer: what is the latest evidence? Curr Oncol Rep 2017;19:67. 28822063 10.1007/s11912-017-0626-1

[28] Nahas R Complementary and alternative medicine approaches to blood pressure reduction: an evidence-based review. Can Fam Physician 2008;54:1529–33. 19005120 PMC2592323

[29] Lee MS Pittler MH Guo R Qigong for hypertension: a systematic review of randomized clinical trials. J Hypertens 2007;25:1525–32. 17620944 10.1097/HJH.0b013e328092ee18

[30] Putiri AL Close JR Lilly HR Qigong exercises for the management of type 2 diabetes mellitus. Medicines (Basel) 2017;4:pii: E59. 10.3390/medicines4030059 PMC5622394 28930273

[31] Freire MD Alves C Therapeutic Chinese exercises (Qigong) in the treatment of type 2 diabetes mellitus: a systematic review. Diabetes Metab Syndr 2013;7:56–9. 23517799 10.1016/j.dsx.2013.02.009

[32] Putiri AL Lovejoy JC Gillham S Psychological effects of Yi Ren Medical Qigong and progressive resistance training in adults with type 2 diabetes mellitus: a randomized controlled pilot study. Altern Ther Health Med 2012;18:30–4. 22516850

[33] Liu T Bai S Zhang RC Effects of Health Qigong Baduanjin on diabetes related indexes in middle-aged obese women. Zhongguo Ying Yong Sheng Li Xue Za Zhi 2018;34:19–22. 29926653 10.12047/j.cjap.5484.2018.006

[34] Elder C Ritenbaugh C Mist S Randomized trial of two mind-body interventions for weight-loss maintenance. J Altern Complement Med 2007;13:67–8. 17309380 10.1089/acm.2006.6237

[35] Xing-Guo S Rehabilitation practice patterns for patients with heart failure: the Asian perspective. Heart Fail Clin 2015;11:95–104. 25432478 10.1016/j.hfc.2014.09.001

[36] Hung HM Chen CH Effects of group qigong exercise on modifying risk factors in adults with ischemic heart disease. J Nurs Healthcare Res 2015;11:277–86.

[37] Moon S Schmidt M Smirnova IV Qigong exercise may reduce serum TNF-α levels and improve sleep in people with Parkinson's disease: a pilot study. Medicines 2017;4:pii: E23. 10.3390/medicines4020023 PMC5590059 28930237

[38] Wu PL Lee M Huang TT Effectiveness of physical activity on patients with depression and Parkinson's disease: a systematic review. PLos One 2017;12:e0181515. 28749970 10.1371/journal.pone.0181515 PMC5531507

[39] Song R Grabowska W Park M The impact of Tai Chi and Qigong mind-body exercises on motor and non-motor function and quality of life in Parkinson's disease: A systematic review and meta-analysis. Parkinsonism Relat Disord 2017;41:3–13. 28602515 10.1016/j.parkreldis.2017.05.019 PMC5618798

[40] Anderson JG Rogers CE Bossen A Mind-body therapies in individuals with dementia: an integrative review. Res Gerontol Nurs 2017;10:288–96. 28981919 10.3928/19404921-20170928-01

[41] Tadros G Ormerod S Dobsonsmyth P The management of behavioural and psychological symptoms of dementia in residential homes: does Tai Chi have any role for people with dementia? Dementia 2013;12:268–79. 24336773 10.1177/1471301211422769

[42] Chan JSM Li A Ng SM Adiponectin potentially contributes to the anti-depressive effects of Baduanjin Qigong exercise in women with chronic fatigue syndrome-like illness. Cell Transplantation 2017;26:493–501. 27938498 10.3727/096368916X694238 PMC5657703

[43] Chan JS Ho RT Wang CW Effects of Qigong exercise on fatigue, anxiety, and depressive symptoms of patients with chronic fatigue syndrome-like illness: a randomized controlled trial. Evid Based Complement Alternat Med 2013;2013:485341. 23983785 10.1155/2013/485341 PMC3747479

[44] Ho RTH Chan JSM Wang CW A randomized controlled trial of qigong exercise on fatigue symptoms, functioning, and telomerase activity in persons with chronic fatigue or chronic fatigue syndrome. Ann Behav Med 2012;44:160–70. 22736201 10.1007/s12160-012-9381-6 PMC3442161

[45] Innes KE Selfe TK Taylor AG Menopause, the metabolic syndrome, and mind-body therapies. Menopause 2015;15:1005–13. 10.1097/01.gme.0b013e318166904e PMC2810543 18779682

[46] Eisenhardt S Fleckenstein J Traditional Chinese medicine valuably augments therapeutic options in the treatment of climacteric syndrome. Arch Gynecol Obstet 2016;294:193–200. 27040419 10.1007/s00404-016-4078-x

[47] Zou L Yeung A Quan X A systematic review and meta-analysis of mindfulness-based (Baduanjin) exercise for alleviating musculoskeletal pain and improving sleep quality in people with chronic diseases. Int J Environ Res Public Health 2018;15:pii: E206. 10.3390/ijerph15020206 PMC5858275 29370149

[48] Mcquade J Prinsloo S Chang DZ Qigong/tai chi for sleep and fatigue in prostate cancer patients undergoing radiotherapy: a randomized controlled trial. Psychooncology 2017;26:1936–43. 27548839 10.1002/pon.4256 PMC5378667

[49] Chan JS Ho RT Chung KF Qigong exercise alleviates fatigue, anxiety, and depressive symptoms, improves sleep quality, and shortens sleep latency in persons with chronic fatigue syndrome-like illness. Evid Based Complement Alternat Med 2014;2014:106048. 25610473 10.1155/2014/106048 PMC4290154

[50] Teut M Knilli J Daus D Qigong or Yoga versus no intervention in older adults with chronic low back pain: a randomized controlled trial. J Pain 2016;17:796–805. 27046802 10.1016/j.jpain.2016.03.003

[51] Yuan Q Guo T Liu L Traditional Chinese medicine for neck pain and low back pain: a systematic review and meta-analysis. PLos One 2015;10:e0117146. 25710765 10.1371/journal.pone.0117146 PMC4339195

[52] Blödt S Pach D Kaster T Qigong versus exercise therapy for chronic low back pain in adults: a randomized controlled non-inferiority trial. Eur J Pain 2015;19:123–31. 24902673 10.1002/ejp.529

[53] Zhang M Xv G Luo C Qigong Yi Jinjing promotes pulmonary function, physical activity, quality of life and emotion regulation self-efficacy in patients with chronic obstructive pulmonary disease: a pilot study. J Altern Complement Med 2016;22:810–7. 27487437 10.1089/acm.2015.0224

[54] Med CMX Med YCZ Efficacy of Liuzijue Qigong in individuals with chronic obstructive pulmonary disease in remission. J Am Geriatr Soc 2015;63:1420–5. 26131612 10.1111/jgs.13478

[55] Chan AW Lee A Lee DT Evaluation of the sustaining effects of Tai Chi Qigong in the sixth month in promoting psychosocial health in COPD patients: a single-blind, randomized controlled trial. ScientificWorldJournal 2013;2013:425082. 24282383 10.1155/2013/425082 PMC3824309

[56] Sawynok J Lynch ME Qigong and fibromyalgia circa 2017. Medicines 2017;4:37. 10.3390/medicines4020037 PMC5590073 28930252

[57] Sawynok J Lynch M Qigong and fibromyalgia: randomized controlled trials and beyond. Evid Based Complement Alternat Med 2014;2014:379715. 25477991 10.1155/2014/379715 PMC4247977

[58] Lauche R Cramer H Häuser W A systematic review and meta-analysis of qigong for the fibromyalgia syndrome. Evid Based Complement Alternat Med 2013;2013:635182. 24288564 10.1155/2013/635182 PMC3833122

[59] Tai HC Chou YS Tzeng IS Effect of Tai Chi synergy T1 exercise on autonomic function, metabolism, and physical fitness of healthy individuals. Evid Based Complement Alternat Med 2018;2018:6351938. 30050592 10.1155/2018/6351938 PMC6040286

[60] Hou PW Fu PK Hsu HC Traditional Chinese medicine in patients with osteoarthritis of the knee. J Tradit Complement Med 2015;5:182–96. 26587390 10.1016/j.jtcme.2015.06.002 PMC4624358

[61] Lee HJ Park HJ Chae Y Tai Chi Qigong for the quality of life of patients with knee osteoarthritis: a pilot, randomized, waiting list controlled trial. Clin Rehab 2009;23:504–11. 10.1177/0269215508101746 19389743

[62] Selfe TK Innes KE Mind-body therapies and osteoarthritis of the knee. Curr Rheumatol Rev 2009;5:204–11. 21151770 10.2174/157339709790192512 PMC3000689

[63] Osypiuk K Thompson E Wayne PM Can Tai Chi and Qigong postures shape our mood? Toward an embodied cognition framework for mind-body research. Front Hum Neurosci 2018;12:174. 29765313 10.3389/fnhum.2018.00174 PMC5938610

[64] Wang CW Chan CL Ho RT The effect of Qigong on depressive and anxiety symptoms: a systematic review and meta-analysis of randomized controlled trials. Evid Based Complement Alternat Med 2013;2013:716094. 23762156 10.1155/2013/716094 PMC3671670

[65] Shamseer L Moher D Clarke M Preferred reporting items for systematic review and meta-analysis protocols (PRISMA-P) 2015: elaboration and explanation. BMJ 2015;349:g7647. 10.1136/bmj.g7647 25555855

[66] Guyatt GH Oxman AD Vist GE GRADE: an emerging consensus on rating quality of evidence and strength of recommendations. BMJ 2008;336:924–6. 18436948 10.1136/bmj.39489.470347.AD PMC2335261

[67] Pieper D Buechter R Jerinic P Overviews of reviews often have limited rigor: a systematic review. J Clin Epidemiol 2012;65:1267–73. 22959594 10.1016/j.jclinepi.2012.06.015

[68] Shea BJ Grimshaw JM Wells GA Development of AMSTAR: a measurement tool to assess the methodological quality of systematic reviews. BMC Med Res Methodol 2007;7:1–7. 17302989 10.1186/1471-2288-7-10 PMC1810543

[69] Lee MS Oh B Ernst E Qigong for healthcare: an overview of systematic reviews. JRSM Short Rep 2011;2:7. 21369525 10.1258/shorts.2010.010091 PMC3046559

[70] Beng-Yeong Ng Qigong-induced mental disorders: a review. Aust N Z J Psychiatry 2015;33:197–206. 10.1046/j.1440-1614.1999.00536.x 10336217

[71] Wayne PM Berkowitz DL Litrownik DE What do we really know about the safety of Tai Chi? A systematic review of adverse event reports in randomized trials. Arch Phys Med Rehabil 2014;95:2470–83. 24878398 10.1016/j.apmr.2014.05.005 PMC4499469

[72] Sancier KM Holman D Commentary: multifaceted health benefits of medical qigong. J Altern Complement Med 2004;10:163–5. 15025890 10.1089/107555304322849084

[73] Khalil H Roughead L Medication safety programs in primary care: a scoping review protocol. JBI Database System Rev Implement Rep 2017;15:1512–7. 10.11124/JBISRIR-2016-003140 28628509

[74] Chan M Wu XY Wu J Safety of acupuncture: overview of systematic reviews. Sci Rep 2017;7:3369. 28611366 10.1038/s41598-017-03272-0 PMC5469776

[75] Xu S Wang L Cooper E Adverse events of acupuncture: a systematic review of case reports. Evid Based Complement Alternat Med 2013;2013:581203. 23573135 10.1155/2013/581203 PMC3616356

[76] Park JE Lee SS Lee MS Adverse events of moxibustion: a systematic review. Complement Ther Med 2010;18:215–23. 21056845 10.1016/j.ctim.2010.07.001

[77] Yin P Gao N Wu J Adverse events of massage therapy in pain-related conditions: a systematic review. Evid Based Complement Alternat Med 2014;2014:480956. 25197310 10.1155/2014/480956 PMC4145795

[78] Aboushanab TS Alsanad S Cupping therapy: an overview from a modern medicine perspective. J Acupunct Meridian Stud 2018;11:83–7. 29436369 10.1016/j.jams.2018.02.001

[79] Klempner SJ Costa DB Wu PA Safety of cupping during bevacizumab therapy. J Altern Complement Med 2013;19:729–31. 23379829 10.1089/acm.2011.0791

